# Pharmacological treatment, adversities, and mobile-based post-discharge follow-up of mucormycosis survivors in Central India: strengthening patient care

**DOI:** 10.3389/frhs.2026.1911647

**Published:** 2026-10-15

**Authors:** Ruchi Baghel, Preeti Acharya, Anshu Verma, Megha Sharma

**Affiliations:** 1 Department of Pharmacology, R. D. Gardi Medical College, Ujjain, Madhya Pradesh, India; 2 Department of Otorhinolaryngology, Government District Hospital, Ujjain, India; 3 Department of Global Public Health, Health Systems and Policy, Medicines (focusing antibiotics), Karolinska Institutet, Stockholm, Sweden

**Keywords:** adverse drug reactions (ADRs), adversities, follow-ups, mobile based post-discharge follow-up, mucomycosis, pharmacological treatment, rhino-orbital cerebral mucormycosis, survivors

## Abstract

**Introduction:**

Mucormycosis, a significant post-COVID-19 complication, is stimulated by uncontrolled diabetes mellitus (DM), prolonged corticosteroid exposure, or immunosuppression. Despite clinical advancements, survivors experience long-term challenges; however, follow-up is often missing from treatment strategies, highlighting a critical gap in safeguarding survivors’ long-term well-being.

**Objective:**

To analyze pharmacological management, adverse events, and post-discharge outcomes among rhino-orbital cerebral mucormycosis (ROCM) patients using medical records and Mobile-Based Post-Discharge (MBPD) follow-up, to inform future clinical and follow-up practices.

**Method:**

Clinical data of confirmed ROCM inpatients (2021) at two COVID-19-dedicated hospitals in Central India were extracted from medical records and analyzed. Mucormycosis survivors were followed up in 2023 using the MBPD questionnaire to explore overall treatment, post-discharge adversities, and outcomes. The clinical findings and patient-reported experiences were summarised descriptively. Factors associated with adversities were examined using multivariable Firth-penalized logistic regression.

**Results:**

Among 108 mucormycosis patients, DM was present in 32% of inpatients. Liposomal amphotericin B (71%) and posaconazole (75%) were predominantly prescribed. *Access* (40%), *Watch* (39%), and Not Recommended antibiotic combinations (13%) were most prescribed. An average of 1.3 adverse drug reactions were recorded among 73 inpatients. Of 97 survivors, 36 were non-responders. Eighty could be contacted, and 61 (76%) consented to participate in MBPD follow-up. Fifty-two of 61 respondents reported 123 post-discharge adversities, averaging 2.4 adversities per patient, including 9 (7%) deaths confirmed by family members. Antifungal prescription was significantly associated with adversities (aOR 5.82, 95% CI 1.56−21.71; *p* = 0.009), whereas older age (>60 years) showed a borderline association. Respondents appreciated the follow-up calls and emphasized regularizing them.

**Conclusion:**

Liposomal amphotericin B, posaconazole, and antibiotics were the most prescribed drugs. DM was the most common comorbidity, highlighting the need for careful monitoring. Antifungal prescriptions were significantly associated with adversities, suggesting the need for implementation of a contextual drug-use policy. The MBPD follow-up was well received and may help monitor recovery, identify health needs, and support the care of survivors of chronic diseases in the future. Survivor-centred care models are suggested to support post-discharge recovery and multidimensional challenges.

## 1 Introduction

The Coronavirus Disease 2019 (COVID-19) pandemic has heavily impacted health systems worldwide, resulting in 5.4 million deaths as reported on 31 December 2021 (1). During the second wave of the COVID-19 pandemic (March to July 2021), the world experienced an exponential rise in a rare fungal infection called mucormycosis, caused by saprophytic fungi of the order Mucorales. State governments in India declared mucormycosis an outbreak in May 2021 (2). In India, mucormycosis affected 0.14 per 1000 people, which was 80 times higher than in high-income countries (3–5). The most common form in India was rhino-orbital-cerebral mucormycosis (ROCM), followed by pulmonary and cutaneous types (5, 6). According to a meta-analysis of 52,916 COVID-19 patients, the incidence of COVID-19-associated mucormycosis (CAM) was 50 times higher (seven cases per 1000 patients) than the highest recorded incidence of mucormycosis worldwide (0.14 cases per 1000 patients) (7). In a report of 275 CAM cases, 85% (233) were from India and 15% from other countries. CAM cases were significantly higher in India, with the report indicating diabetes mellitus (DM) as the most common underlying risk factor in India compared to other nations (8). Additionally, widespread corticosteroid use, environmental exposure to fungal spores, and uneven access to healthcare contributed to the surge. Another study of 99 CAM patients found that 72% were from India, most patients (78%) were male, and 85% had DM. A history of COVID-19 was noted in 37% of these patients who developed mucormycosis after recovery (9). Mucormycosis mortality in India was 50% before the COVID-19 pandemic and rose to 85% during the pandemic, drawing global attention (10). A multicentric, multistate Indian study of 2826 ROCM patients reported 73% with regressed ROCM (11). However, the study primarily included ophthalmologic facilities, with only 10% of total patients from 3 cities of Madhya Pradesh and a short follow-up period (mean 14 days), indicating potential under-reporting (11).

The high number of CAM cases and the rising prevalence of DM in India emphasize the need for thorough investigation (12). However, CAM cases may still be underreported. Underreporting likely due to limited diagnostic resources, inconsistent reporting, variable clinical recognition, and stigma associated with the disease. This underreporting is concerning because it hides the actual burden and hinders public health efforts to anticipate future outbreaks. Effective mucormycosis management involves rapid control of risk factors, antifungal therapy, surgical removal of infected tissue, and supportive care (13).

The mucormycosis treatment guidelines recommend liposomal amphotericin B (14). Although the sudden incursion of mucormycosis led to an increase in the use of multiple antifungal agents, most prominently amphotericin B deoxycholate, liposomal amphotericin B, and oral posaconazole, and a combination of liposomal amphotericin B with newer antifungals like posaconazole and isavuconazole (11, 15). However, combination therapy did not show improved outcomes compared with monotherapy in a propensity score analysis of 106 patients with hematologic malignancies (16). Moreover, the combination therapy has resulted in an unavoidable increase in the incidence of adversities, including adverse drug reactions (ADRs) (17).

The world was not prepared to tackle the COVID-19 pandemic and associated mucormycosis; therefore, it is important to study how it was addressed to be future-ready. However, complete data are not available (severe under-reporting); yet the available studies show that survivors of mucormycosis often face long-term sequelae, including facial deformities, neurological deficits, and psychological impacts, which adversely affect their quality-of-life and ability to resume normal activities (18). Therefore, understanding in-hospital and post-discharge care is also essential to plan for future pandemic-related side effects. Nonetheless, despite their crucial role, post-discharge follow-ups are often not a part of treatment guidelines. This highlights a critical gap in upholding survivors’ health status and must be addressed urgently.

Therefore, this study investigated the clinical and pharmacological management of patients diagnosed with ROCM (mucormycosis) admitted to two COVID-19-dedicated hospitals (public and private) in Central India and explored post-discharge quality-of-life outcomes using a Mobile-Based Post-Discharge (MBPD) follow-up. The overarching goal was to generate evidence to strengthen continuity of care, inform prevention strategies, and improve long-term outcomes for patients with mucormycosis and similar complex conditions.

## 2 Methodology

This study was reported in accordance with the Strengthening the Reporting of Observational Studies in Epidemiology (STROBE) statement (19).

### 2.1 Study design and population

An ambispective design was used to achieve the study aim, integrating quantitative descriptive analyses of medical records with MBPD follow-up using a structured questionnaire, as explained below. Medical records of all patients admitted to the study hospitals during the study period were screened.

**Inclusion Criteria:** The cohort included patients diagnosed with biopsy-proven mucormycosis of the paranasal sinuses, orbit, or infratemporal fossa (ROCM) aged >18 years. Incomplete data were attempted to be retrieved from archived patient records. If particular information was unavailable, we removed that patient's record from the respective variable analyses, resulting in variable-dependent denominators across analyses. However, this was crucial to overcome under-reporting. **Exclusion Criteria:** Patients below 18 years old and those with duplicate or incomplete records were excluded from the retrospective part of the study. Patients who did not answer after 10 calling attempts, had incorrect or incomplete phone numbers in the medical records, or did not consent to participate were excluded from the MBPD follow-up study.

### 2.2 Data collection: tools, validation and process

The information on clinical and diagnostic management of mucormycosis was extracted from patients’ medical records and entered into Microsoft Excel. The MBPD questionnaire was developed as a structured instrument with 27 closed-ended questions and one open-ended question to record respondents’ additional comments and experiences (Supplementary material 1). The question guide was validated through pilot tests with patients discharged after other surgical procedures (data not included in this study) and modified based on feedback. It included questions on drugs administered; post-discharge adversities, including mortality; post-discharge functional issues; and treatment outcomes such as well-being, disease-specific issues such as anxiety, visual loss, difficulty speaking or performing daily routines, and any comorbidities, experienced discomfort, or conditions related to the treatment.

#### 2.2.1 Patient's medical records

Medical records of confirmed mucormycosis patients admitted between May and July 2021 at the study hospitals were selected for screening. Figure 1 shows the details of patient selection, screening, and inclusion for both the retrospective and prospective parts of the study. Information extracted from mucormycosis patients’ medical records included demographics, comorbidities, drugs administered and duration, laboratory findings, length of stay (LOS), whether a surgical procedure was performed, and hospital admission outcome. The antifungal and antibiotic prescriptions were reviewed using WHO recommendations, including the AWaRe classification and antimicrobial stewardship, to assess adherence and the justification for prescribing drugs (20).

**Figure 1:**
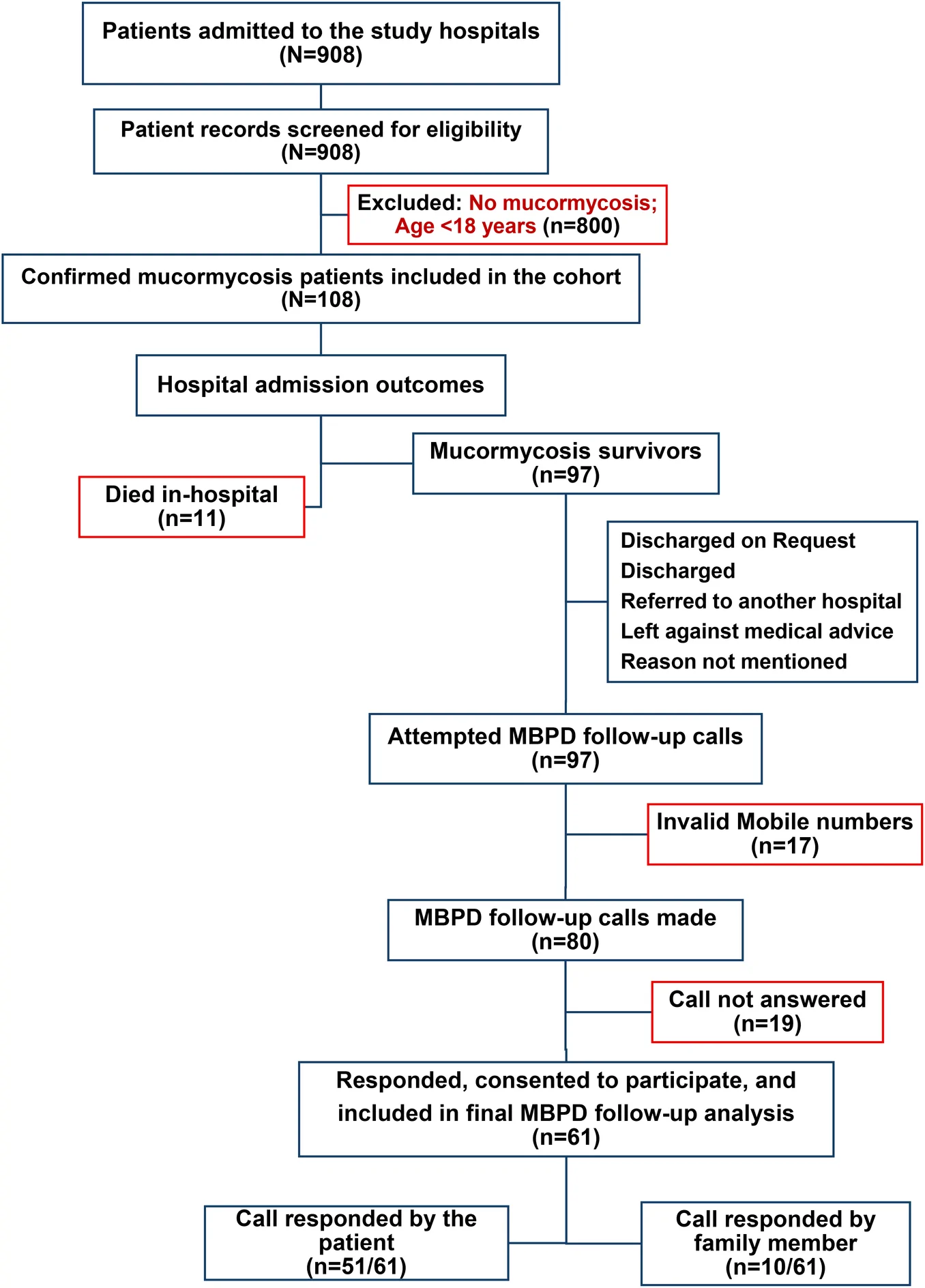
STROBE-compliant flow chart of the mucormycosis cohort from admission at two COVID-19 dedicated hospitals in Central India to MBPD follow-up. The red boxes represent patients excluded from the analyses (No mucormycosis, Age <18 years, incomplete records, invalid phone number, no response to the calls).

#### 2.2.2 The mobile-based post-discharge follow-up

The MBPD follow-up questionnaire was developed in *Hindi,* translated into English, and back-translated to confirm the content reliability. A pharmacologist, public health expert, epidemiologist, and otorhinolaryngologist drafted and finalized it by consensus. MS, an experienced qualitative researcher, trained the interviewer (PA) on the interview guide and objectives before data collection. MBPD follow-up telephonic interviews were conducted prospectively between January and March 2023, targeting mucormycosis survivors (i.e., patients who left the hospital alive).

Information obtained through MBPD follow-up calls was transcribed verbatim. Frequently reported words and phrases were identified and entered into a structured Excel sheet. Those were then grouped. and summarised anonymously using descriptive statistics to provide an overview of participant compliance and reported outcomes.

### 2.3 Operational definitions

**Mucormycosis:** Mucormycosis is a rare, aggressive, life-threatening, and angio-invasive fungal infection caused by mold of the order *Mucorales,* omnipresent in indoor and outdoor environments (21). The most common type is ROCM. ROCM in our study was confirmed as per ISHAM recommendations, i.e., when clinical features and radiological findings (contrast-enhanced magnetic resonance imaging and computed tomography scan) were consistent with the diagnostic parameters, and fungal elements were demonstrated in tissue samples by potassium hydroxide mount, culture, and/or histopathological examination (21).

**Adversity:** Any new or reported persistent symptoms, complications, or functional impairments experienced following treatment for mucormycosis, irrespective of whether a causal relationship with the disease or its treatment could be established.

**AWaRe:** The WHO AWaRe classification categorizes antibiotics into: *Access* (first-line), *Watch* (requiring careful monitoring), and *Reserve* (last-resort) categories, based on their spectrum of activity, potential for selecting antimicrobial resistance, and recommended role in clinical practice (20).

### 2.4 Data analysis

Analyses were conducted using Stata SE version 16.1 and Microsoft Excel (22). Descriptive statistics were used to summarize demographic, clinical, and treatment characteristics. Continuous variables are presented as means with ranges or medians with interquartile (IQR) ranges, and categorical variables as frequencies and percentages. Percentages were rounded to the nearest whole number, thereby sometimes resulting in values below or above 100%.

The study identified ADRs based on deranged laboratory parameters. Statistical significance was set at *p* < 0.05. Causality analysis of ADRs was conducted using the WHO causality scale, and severity was assessed using Hartwig and Siegel's scale of severity assessment (23, 24). Responses from MBPD follow-up interviews were transcribed verbatim in Microsoft Word and grouped using keywords. Frequently reported words, phrases, and issues were identified, grouped (for example, adversity experienced, diabetes developed after discharge, amphotericin was prescribed, financial issues were experienced: Yes/ No, number of days of stay, treatment), and entered into the structured Microsoft Excel sheet. This provided contextual information to complement the quantitative results rather than as a standalone qualitative analysis. Therefore, MBPD follow-up responses were analyzed descriptively using percentages and frequencies. If the respondent reported any adversity or the patient was unwell at follow-up, the patient was advised to visit the nearest healthcare facility/hospital.

For this ambispective study of mucormycosis patients, the sample size was determined by the number of eligible patients (*N* = 108). Therefore, we did not perform a formal *a priori* power calculation. Given the relatively modest cohort size and sparse outcome distribution, a Firth-penalized logistic regression model was used to examine factors associated with adversity (yes/no), antifungal prescription, gender, and age groups. The predictors were selected based on clinical relevance. Odds ratios (ORs) and 95% confidence intervals (CIs) were derived from the penalized likelihood model.

### 2.4 Ethical considerations

The Ethics Committee of the R. D. Gardi Medical College approved the study with approval number 19/2022. The study extracted pharmacological treatment data from patients’ medical records and involved no direct interaction with patients. For the MBPD follow-up, the purpose of the call was explained to the interviewee, and verbal consent was obtained from respondents to participate and to record the interview for future reference. The data were anonymised, and all identifiable variables were replaced with codes before analysis. Only the authors have access to the data files stored on a password-protected computer, ensuring full confidentiality.

## 3 Results

### 3.1 Patients’ profile and pharmacological management

Overall, 908 patients were admitted to the study hospitals during the study period. Of these, 12% of patients (108/908) had a confirmed diagnosis of mucormycosis and were included in the analysis. The median LOS for 108 patients was 14 days (IQR: 6−25, range: 1 to 89 days). Most inpatients were male (64%) and aged 40−60 years (66%, Table 1). More than half of the patients were admitted to the private-sector hospital (67%). Of all patients, 25% were discharged, 24% were referred to other hospitals, 29% were discharged on request, and 10% died during their hospital stay (Table 1).

**Table 1:** Demographic parameters and length of stay Among mucormycosis patients as per patients’ medical records at two COVID-19 dedicated hospitals in Central India.

Variables	Number of patients with confirmed mucormycosis (*N* = 108)	Percentage
Type of hospital	**n**	
Private-sector	72	67
Public-sector	36	33
Gender		
Male	69	64
Female	39	36
Age group		
18–39	20	19
40–60	71	66
>60	17	16
Length of stay		
≤7 Days	31	29
8–14 Days	23	21
15–30 Days	41	38
>31 Days	13	12
Length of hospital stay in days: Median (IQR) [Mean ± SD]	14 (6-25) [17 ± 13.54]	
Outcome of admission		
Discharged on request	31	29
Discharged	27	25
Referred to a Public Hospital	19	18
Died	11	10
Left against medical advice	8	8
Referred to a private hospital	7	6
Not specified in patient record	5	5

*N* = total study population. *n* = number of patients with data available for the specific variable. Values presented as Median (IQR) [Mean ± SD], or *n* (%), SD, standard deviation; IQR, Interquartile range. Percentages are rounded to the nearest whole number.

Of 108 patients, 90 (83%) received at least one antifungal, of whom 71% received liposomal amphotericin B, 75% received posaconazole, and 18% received voriconazole. Notably, 8 patients received only liposomal amphotericin B, whereas 12 received only posaconazole. Sixty-nine patients received concomitant treatment of liposomal amphotericin B and posaconazole. The mean duration of amphotericin B and posaconazole prescriptions was 16 days. A combination of two or more antifungals was prescribed to 64% of patients (69/108). Other commonly prescribed drugs were insulin in 46% of patients and steroids in 10%. Apart from these drugs, ranitidine and its FDCs with various vitamins and minerals were prescribed to 56% of patients. Anticoagulants were prescribed to 14% of patients (Table 2).

**Table 2:** Duration of prescribing antifungals and other drugs among mucormycosis inpatients at two COVID-19 dedicated hospitals in Central India.

Antifungals	Patients N (%)	Mean (Days)	Range in days
*Total N (%)*	**108** (**100)**		
Posaconazole	81 (75)	16	3–39
Liposomal Amphotericin B	77 (71)	16	1- 58
Voriconazole	19 (18)	6	2–14
Other drugs			
Ranitidine and its FDCs	60 (56)	16	1–58
Anticoagulants	15 (14)	14	1–58
Insulin	50 (46)	14	1–58
Steroids	11 (10)	6	1- 9
Antiplatelets	7 (7)	10	2–20

FDCs, fixed dose combinations. *N* = total study population. *n* = number of patients with data available for the specific variable. Percentages are rounded to the nearest whole number. The bold fonts represent the total number of observations used as the denominator for the variable.

All ADRs were identified by active surveillance of medical records, as no spontaneous ADRs were reported to the ADR Monitoring Center during this period. Among 108 inpatients, 68% (73/108) had deranged laboratory parameters. Hyponatremia was observed in 50/108 patients (46%) and hypokalemia in 47/108 (44%). Overall, 97 ADRs were observed in 73 patients with an average of 1.3 ADRs per patient. All recorded ADRs were categorized as ‘possible’ according to the WHO causality assessment scale. Based on the modified Hartwig and Siegel severity assessment scale, 72% of ADRs were mild, and 28% were moderate. In addition, the lactate dehydrogenase and alanine aminotransferase levels were elevated in 25% each (19 inpatients).

The study population received an average of two antibiotics per patient. Almost half of the antibiotics (46%) were administered parenterally. Ceftriaxone and its fixed-dose combinations (FDCs) with sulbactam were the most prescribed antibiotics. *Access* antibiotics were prescribed most frequently (40%), followed by *Watch* (39%) and *Reserve* antibiotics (8%). Not recommended FDCs were prescribed in 13% of occasions (Table 3).

**Table 3:** Antibiotics prescribed to mucormycosis patients at two COVID-19 dedicated hospitals in Central India: categorized by route of administration and the WHO's AWaRe classification.

Class and name of the drug	Prescription frequency N (%)	Route of administration, *AWaRe* categories
*Antibiotic substances*	**153 (100)**	
Ceftriaxone	29 (19)	IV, *Watch*
Metronidazole	28 (18)	PO, *Access*
Amoxicillin + Clavulanic acid	24 (16)	PO, *Access*
Ceftriaxone + Sulbactam	19 (12)	IV, Not recommended
Ciprofloxacin	16 (11)	PO, *Watch*
Meropenem	12 (8)	IV, *Reserve*
Doxycycline	9 (6)	PO, *Access*
Moxifloxacin	8 (5)	IV, *Watch*
Azithromycin	2	PO, *Watch*
Cefpodoxime	1	PO, *Watch*
Cefuroxime	1	PO, *Watch*
Cefoperazone + Sulbactum	1	IV, Not recommended
Linezolid	1	PO, *Reserve*
Vancomycin	1	IV, *Watch*
Piperacillin + Tazobactum	1	IV, *Watch*

IV, intravenous; PO, oral route. Percentages are rounded to the nearest whole number. The bold fonts represent the total number of observations used as the denominator for the variable.

### 3.2 Comorbidities, and surgical management

Among 108 patients, 42% had a history of COVID-19 infection (Table 4). DM or DM with other comorbidities was present in 32% of patients, and 14% had a history of steroid use prior to admission. All ROCM patients included in this study underwent endoscopic surgical debridement, i.e., Denker's and Modified Denker's surgery, with orbital exenteration in 23% of cases. All cases were thoroughly screened daily with endoscopic clearance for recurrence of mucor in the paranasal sinuses and orbit, with extension into the infratemporal fossa and suspected frontal region. Renal function tests and serum potassium were also monitored daily.

**Table 4:** Comorbidities among mucormycosis patients treated at the study hospital in Ujjain district of Central India.

Variables	Number of patients	Percentage
	*N* = 108	100%
COVID-19 infection (as per patient record)		
No history	48	44
H/O Covid-19	46	42
Covid-19 positive	7	6
Covid-19 suspected	4	4
Covid-19 negative with B/L pneumonia	3	3
*Endoscopic surgical debridement*	**108**	**100**
*Total diabetes mellitus (DM)*	**35**	**32**
DM	11	10
Post-COVID DM	6	5
DM and hypertension (HTN)	9	8
DM, HTN and asthma/cardiovascular disease/hemiparesis	9	8

All percentages are calculated with a denominator of 108. Percentages are rounded to the nearest whole number. The bold fonts represent the total number of observations used as the denominator for the variable.

### 3.3 Mobile-based post-discharge follow-ups

Out of 108 patients, 11 died in the hospital during treatment; therefore, attempts were made for MBPD follow-up to the remaining 97 survivors using mobile numbers recorded in the patient records. Of these, 17 (18%) numbers were incomplete or incorrect (Figure 1). The remaining 80 calls connected, and the caller tune was heard, but 19/80 (24%) did not answer. Therefore, 36/97 survivors could not be contacted even after repeated attempts. Of total 80 MBPD follow-up calls made, 61/80 respondents received the call and consented to participate in the study, leading to 76% response rate (Table 5). Among the respondents, 51 were patients and 10 calls were responded by family members of which 9 patients had died. Most of the respondents were male (71%). The mean call duration was 7.33 min (range 4–15 min).

**Table 5:** Summary of reported responses during mobile-based post-discharge follow-ups .

Response to follow-up call	Frequency	Percentage
*Total MBPD follow-up calls made*	**80**	**100**
Call not answered	19/80	24
Responded to the MBPD call and consented to participate	61/80	76
*Total MBPD calls responded*	**61**	
Calls responded by patients	51/61	84
Male	35/51	69
Female	16/51	31
Call responded by family members	10/61	16
Male	8/10	80
Female	2/10	20
*Medical history as reported by the responders	**61**	**100**
H/o COVID-19 (*N* = 61)	42	69
H/o steroid administration	28	46
H/o oxygen use	19	31
H/o remdesivir administration	13	21
COVID-19 treatment was received at-		
Private sector hospital	22	36
Home isolation	20	33
Public sector hospital	10	16
No history of COVID-19	9	15
*Healthcare facilities utilized for mucormycosis treatment*		
Private sector facility	43	71
Public sector facility	18	29
*Reported length of hospital stay for mucormycosis in days:* *Median (IQR) [Mean ± SD]*	30 (22–48) [37.5 ± 26.3]	
*Reported days between the development of mucormycosis post-COVID-19:* *Median (IQR) [Mean ± SD]*	4 (0–8) [7.6 ± 16.54]	
Reported post-discharge adversities and outcomes		
No issue reported	9	15
*Total adversities reported by 52 respondents*	**123**	**100**
Hypokalemia	27	22
Vomiting	16	13
Nausea	14	11
Death after discharge	9	7
Diabetes mellitus developed after receiving mucormycosis treatment	9	7
Loss of vision	9	7
Eye pain	7	6
Nephrotoxicity	2	2
Headache	6	5
Loss of sensation	4	3
Swelling on face	4	3
Hypertension	3	2
Difficulty in breathing	2	2
Loose teeth	2	2
Memory loss	2	2
Other issues^#^	7	6

Other issues^#^ =Heart attack post-discharge (1), pain in upper jaw (1), mouth pain (1), hypertension (1), delayed wound healing after treatment (1), pedal edema after discharge (1), allergic reaction after treatment (1).*Medical History as reported by the responders: Each variable is individual; therefore, the calculated percentages are for respective variables. H/O, History of. LOS; Length of stay in hospital.

Percentages are rounded to the nearest whole number. The bold fonts represent the total number of observations used as the denominator for the variable.

Out of 61 respondents, 69% of patients reported a history of COVID-19 infection, and 46% reported a history of steroid administration. A history of remdesivir administration was reported by 13 patients, and 62% expressed experience of adverse effects. Most of the patients were treated in private-sector hospitals for COVID (36%) and mucormycosis (71%; Table 5). The median duration for the development of mucormycosis after COVID-19 infection was 4 days (IQR: 0–8; range: 0−120 days).

Of the total 61 respondents, 52 (85%) experienced 123 post-discharge adversities, including the death of 9 patients, averaging 2.4 adversities per patient. The most frequently reported post-discharge adversities included hypokalemia (22%), vomiting (13%), and new-onset diabetes following treatment in 7% of patients (Table 5). Six responders reported financial burden due to medical expenses. Survivors and family members appreciated the follow-up calls; they felt surprised and emphasized the need for regular follow-up, support, and care as evident from the following quotes.

“This call felt like someone cares about us. It was devastating to lose my mother and have no one to talk to.” (Family member of patient-3)

“You are asking about my father! My father has been in the best of health since last year (sarcastic). He must be watching from heaven that someone is asking about him. He must be feeling special as I do.” (Family member of patient-9)

“If the government starts a scheme to call and follow-up the patients with serious illness, this will give a sense of being cared, and for the patient who is unable to go to a doctor, he can get help from such calls.” (Patient-31)

I never thought someone would call me to ask, “How am I feeling now?” Those were days of bad dreams. I am alive, and I am happy for that. Thank you for this call.” (Patient-49)

Table 6 shows a comparative presentation of the major outcomes of mucormycosis patients based on information provided in medical records and self-reported through MBPF follow-up.

**Table 6:** Comparative presentation of the major outcomes of mucormycosis inpatients based on medical records and MBPD follow-up.

Variables	As per patient's medical records N = 108 n (%)	As per MBPD follow-up N = 61 n (%)
Liposomal amphotericin B prescribed	77 (71)	58 (95)
Diabetes mellitus	35 (32)	9- New onset (15)
COVID-19 infection present	57 (53)	41 (67)
LOS median (IQR)	14 (6–25)	30 (22–48)
Deaths	11 (10)	9 (15)
Average adversities	1.25^a^	2.4

a ADRs were considered adversities for hospitalised patients. Total entries for hospital patient records were *N* = 108; total entries for MBPD follow-up calls were *N* = 61; *n* = number of patients/respondents for the specific variable. All percentages are rounded to the nearest whole number.

LOS, length of stay in hospital; MBPD, mobile based post-discharge.

In the multivariable Firth-penalized logistic regression analysis, receipt of any antifungal prescription was independently associated with increased odds of adversity (Table 7, OR 5.82, 95% CI 1.56−21.71, *p* = 0.009). Patients aged more than 60 years had higher odds of adversity compared with those aged 18−39 years (OR 3.95, 95% CI 0.94−16.51, *p* = 0.060), while those aged 40−60 years had 2.40-fold higher odds (OR 2.40, 95% CI 0.85−6.74, *p* = 0.097). Females tended to have lower odds of adversity than males (OR 0.47, 95% CI 0.20−1.12, *p* = 0.090), although the association was not statistically significant. Overall, the model was statistically significant (Wald *χ*^2^ = 12.83, *p* = 0.012).

**Table 7:** Factors associated with adverse outcomes: multivariable firth-penalized logistic regression.

Variable	aOR	95% CI	*p*-value
Age group			
18–39 years (Ref)	1	-	-
40–60 years	2.4	0.85–6.74	0.097
>60 years	3.95	0.94–16.51	0.06
Gender			
Female (Ref)	1	-	-
Male	0.47	0.20–1.12	0.09
Any antifungal prescribed			
No (Ref)	1	-	-
Yes	5.82	1.56–21.71	0.009

aOR, adjusted odds ratio; CI, confidence interval. Odds ratios were estimated using the Firth-penalized logistic regression. (Ref)- Reference categories were age 18–39 years, male gender, and no antifungal prescription.

## 4 Discussion

Healthcare delivery in Central India faces several challenges and is scarcely represented in the existing literature. Our study focuses on patients diagnosed with mucormycosis from hospitals in Central India, a region where related evidence is lacking. This study provides valuable insights into drug selection, clinical and surgical outcomes, and the long-term effects of therapy for this severe, life-threatening fungal infection. Most inpatients were male (64%) and aged 40−60 years (66%). The present study identified post-discharge health concerns, including mortalities among mucormycosis survivors that likely would have gone unreported without our MBPD follow-up.

The gender distribution (64% males) in our cohort aligns with previously reported skewness worldwide, with males having a higher incidence and poorer outcomes for mucormycosis (25–27). This demographic skew may also affect follow-up engagement and outcomes, underscoring the need for additional gender-sensitive, region-specific approaches in post-infection care. Conducting this study in Central India was crucial, as the region faces rising diabetes prevalence and resource constraints that limit access to medical care (12). Our study offers insights for clinicians and policymakers to improve future clinical practice and bridges a major evidence gap in similar settings.

### 4.1 Pharmacological treatment in medical records

In the present study, the disease imposed a significant burden on healthcare resources, with a median LOS of 14 days and a mortality of 10%. However, the mean LOS in other available studies was longer. For example, an Indian study on mucormycosis patients reported an LOS of 22 ± 6 days and a mortality of 18% (27); a United States nationwide study reported an LOS of 24.5 days with 30% mortality (28); and an Egyptian study reported a mortality of 49% (29). The shorter LOS in our study may reflect the rhino-orbital disease spectrum and timely intervention; however, under-reporting cannot be ruled out (30, 31). Additionally, about a quarter of patients were referred to other facilities, suggesting treatment may have continued there (25).

Another notable finding was the reported duration of mucormycosis development after COVID-19, with a median of 4 days after COVID-19 infection and a significant proportion of patients having a history of COVID-19 infection (42 respondents). This was significantly lower than previously reported 15 to 22 days (7). Also, all patients in our study underwent surgical procedures, including orbital and sinus debridement; similar results were reported in an Indian study by Karat S et al. (32). However, the short duration of mucormycosis development and the surgical procedures performed suggest the possibility of more severe or worsening conditions in our cohort, but the possibility of recall bias cannot be ignored; thus, this requires further investigation.

The rapid increase in mucormycosis incidence during the COVID-19 pandemic was observed globally and was largely attributed to factors such as prolonged steroid use, uncontrolled diabetes, and immunodysregulation (10, 12, 33, 34). However, the low percentage of patients with a history of steroid intake in our study (10%) suggests that diabetes might have played a significant role in the pathogenesis of mucormycosis in this cohort (34, 35). Multicentric studies also support that diabetes cases are at risk and are increasing rapidly in Central India (11, 34). The high prevalence of DM (32%) among the patients underscores the critical role of hyperglycemia in predisposing individuals to mucormycosis. Diabetes-related immune dysfunction creates a favorable environment for *Mucorales* fungi to grow (33, 34).

The average prescription of two antibiotics per patient suggests a high clinical suspicion of secondary bacterial infections rather than microbiologically confirmed coinfections. Patients with mucormycosis frequently have risk factors such as diabetes, prolonged hospitalization, prior steroid use, and invasive procedures, which increase vulnerability to bacterial superinfections; however, documented bacterial coinfection rates remain variable and often lower for empiric antibiotic use (35). Studies show that antibiotics are often prescribed based on clinical deterioration, fever, or radiological findings, even without culture-proven bacterial infection, highlighting the need for effective antimicrobial stewardship (35). The predominance of parenteral administration (48%) indicates that intravenous antibiotics are preferred for their proven efficacy in severe infections.

*Access* antibiotics constituted the largest proportion of antibiotic prescriptions (40%); however, this was substantially below the WHO target of using at least 60% of antibiotics from the *Access* category (36, 37). The relatively high use of *Watch* and *Reserve* antibiotics is a concern from an antimicrobial stewardship perspective, as these categories have greater potential to drive antimicrobial resistance and should be used judiciously. Moreover, the frequent prescription of ceftriaxone and its fixed-dose combination with sulbactam, as observed in the present study, reflects the empirical use of broad-spectrum antibiotics in patients with mucormycosis (37). In COVID-19–associated mucormycosis, broad-spectrum *β*-lactams were commonly initiated to cover suspected gram-negative and mixed infections until definitive microbiological results are available. However, these prescribing patterns are not reported from India or global cohorts during the post-COVID surge of mucormycosis, which is concerning.

The use of WHO “Not Recommended” fixed-dose combinations (13%) is particularly noteworthy, as these combinations lack evidence-based indications or recommendations in international guidelines. Their continued use represents a persistent prescribing concern, particularly in India, where substantial use of WHO Not Recommended antibiotic combinations has previously been documented (38). However, in addition to surgical procedures, the exceptional circumstances of the COVID-19 pandemic, including diagnostic uncertainty, high patient burden, and empirical treatment of suspected secondary infections, may have contributed to such prescribing practices. These observations highlight the need for pandemic-specific antimicrobial stewardship policies and prescribing protocols to promote rational antibiotic use during future public health emergencies and minimize the potential emergence and spread of antimicrobial resistance.

Liposomal amphotericin B is the drug of choice due to its broad-spectrum activity against *Mucorales* and proven efficacy in reducing mortality when initiated early in the disease course. However, because of supply shortages during the COVID-19 surge and the nephrotoxicity associated with amphotericin formulations, posaconazole was often used as an alternative or as step-down therapy once clinical stabilization was achieved (39). The high use of antifungal agents, particularly liposomal amphotericin B (71%) and posaconazole (75%), reflects adherence to the standard treatment protocols for mucormycosis (39, 40).

A subset of patients in our cohort received monotherapy with either liposomal amphotericin B or posaconazole, which reflects real-world treatment constraints rather than evidence-based preference. Although posaconazole and isavuconazole are recognized salvage options, current global and national guidelines do not recommend concurrent use of two systemic antifungal agents for mucormycosis because robust clinical data supporting combination therapy are lacking (39–42). Therefore, treatment decisions were primarily guided by drug availability, patient tolerance, and evolving consensus during the pandemic period. The mean duration of treatment with these antifungals was 16 days, consistent with recommendations for prolonged therapy to achieve a favourable therapeutic outcome (27, 39).

Electrolyte imbalances were common during antifungal therapy, with hyponatremia and hypokalemia being the most frequent findings. Hypokalemia is a well-recognized adverse effect of liposomal amphotericin B (43) and has also been reported with posaconazole, along with hepatic enzyme elevations, including ALT (44). Hyponatremia, although frequently observed in our cohort, is less specifically associated with these antifungal agents and may be multifactorial, particularly in severely ill and hospitalized patients. As both antifungal agents were administered concomitantly, these laboratory abnormalities cannot be attributed to either drug individually, emphasizing the importance of monitoring electrolytes and liver function during antifungal therapy.

All 97 ADRs were classified as ‘possible’ because limited clinical information and concomitant polypharmacy in the records made it difficult to establish a definite causal relationship with individual drugs. Most reported adverse events in our study were mild or moderate; however, incomplete clinical information on medication modification in the patient's records limits the assessment. Physicians may have focused primarily on managing patients under the restricted and demanding conditions of the COVID-19 pandemic. A study by Tiwari and Vakil reported that all antifungal-related ADRs in patients with COVID-associated mucormycosis were mild (32%) and moderate (68%), with no severe ADRs observed (45). However, the proportion of mild and moderate ADRs differed from that in our study.

Ranitidine was frequently prescribed in our study despite international concerns regarding N-nitrosodimethylamine (NDMA) contamination. Ranitidine was withdrawn from the US market and suspended in the European Union in 2020 because of potential exposure to unacceptable levels of NDMA. Ranitidine was removed from the National list of Essential Medicines but was not banned in India (46). Indian regulatory authorities recommended testing and monitoring NDMA levels in marketed formulations. Thus, its continued use in our cohort during 2021 may reflect the differing regulatory position and prescribing practices in India at that time (47). These findings support the urgency for the development of appropriate drug-safety policies and regulatory recommendations regarding the use of ranitidine in India.

### 4.2 Mobile-based post-discharge follow-ups

Our study provides important insights into post-discharge outcomes among patients with a history of mucormycosis, a topic that remains underexplored. Of the 97 survivors, 80 (82%) were reachable by phone, and 61/80 (76%) responded, reflecting a strong engagement rate that overcame the challenges of long-term surveillance in high-morbidity cohorts. The response distribution, with 84% from living patients and 16% from family members, underscores the value of including proxy respondents in mortality and morbidity monitoring in high-risk infections (48).

The reported post-discharge mortality of 15% (9/61) underscores the ongoing vulnerability of this population even after hospital discharge. These findings echo the necessity of routine integration of long-term follow-up in mucormycosis cohorts, which emphasize the persistent risk of death and complications despite initial survival. For example, a multicentric cohort study found that among hospitalized patients with mucormycosis, predictors of mortality extended into the post-hospital period (49). In addition, in our study, the MBPD follow-up does not include 36/97 patients who survived having incomplete or wrong phone numbers or not answered to the call. This might lead to overly optimistic representation of health and functional outcomes, experiences of reported adversities, as the survival status of 36 non-respondents was unknown. Therefore, the post-discharge follow-up is recommended. That can play a crucial role in supporting survivors’ long-term outcomes, particularly for significant medical conditions such as mucormycosis.

The mean call duration of 7.33 min suggests that the MBPD follow-up was operationally feasible and efficient. However, the non-response rate (24%) suggests plausible barriers to complete data capture, such as stigma, grief, communication fatigue, or difficulties with contact tracing due to recording errors, incomplete numbers, or mishearing figures while filling out the patient file (18, 49). More importantly, the possibility of post-discharge death of the survivor cannot be ruled out, thereby resulting in underreporting of deaths from mucormycosis or associated comorbidities. This suggests the need for alternative ways to contact patients with such severe illness while planning for such follow-ups.

Notably, 69% of respondents reported a history of concurrent COVID-19 and mucormycosis in our study. Although the primary focus of this analysis is on mucormycosis outcomes, the high rate of co-infection in our cohort reflects the known surge in CAM during the second wave, particularly among individuals with diabetes and those exposed to steroids. Thus, while the index event here is mucormycosis, the antecedent COVID-19 may remain an important contextual risk factor. The high prevalence in our cohort reinforces the need for vigilant screening and early antifungal intervention in high-risk COVID-19 patients (50).

Remdesivir exposure was reported in 13 patients (21%), of whom 8 (62%) experienced adverse effects. Although remdesivir has been shown to shorten recovery time in hospitalized patients, its safety profile remains under scrutiny (51). Our findings echo these concerns and support cautious use, close monitoring, and post-discharge pharmacovigilance.

Most of the inpatients received treatment for both mucormycosis and COVID-19 at the private-sector hospital. However, hospital type was not a concern during the pandemic because both study hospitals were dedicated COVID-19 hospitals; therefore, the government provided antifungal drugs free of cost to both hospitals. As a result, the medicine supply may have supported optimal care for affected patients without an additional financial burden. Yet, six patients reported financial burden during MBPD follow-up calls, indicating that economic pressure on families cannot be disregarded.

Another interesting finding was the discrepancy between self-reported and documented LOS in hospital (Table 6), where the LOS reported during MBPD follow-up was twice as high as in the medical records. This may be because a quarter of inpatients were not discharged but were referred to other hospitals to continue treatment, reflecting disease severity. Moreover, follow-up occurred about two years after treatment; therefore, recall bias over this prolonged interval must also be considered. While telephone follow-up remains a feasible approach for assessing long-term outcomes, caution is warranted when interpreting patient-reported details that are susceptible to imperfect recall.

In the Firth-penalized logistic regression analysis, receipt of any antifungal prescription was independently associated with nearly six-fold higher odds of adversity. This finding is consistent with the pharmacodynamic profile of systemic antifungal agents, including hepatotoxicity, nephrotoxicity, gastrointestinal adverse effects, and drug-drug interactions, which may necessitate treatment modification or discontinuation (52, 53).

Older age was associated with higher odds of adversity, with patients aged >60 years showing almost four times more likely to experience it than younger adults, although the association was not statistically significant. Yet, the observed trend is biologically plausible given age-related physiological changes, multimorbidity, polypharmacy, and altered pharmacokinetics and pharmacodynamics, which increase susceptibility to adverse outcomes (54). Female patients had lower odds of adversity than males; however, the association needs cautious interpretation.

The overall model was statistically significant, suggesting that the included covariates collectively explained variations in adversity occurrence, while Firth penalization enhanced estimate stability in the presence of sparse outcome data.

Moreover, metabolic comorbidities such as diabetes mellitus have been identified as independent risk factors that amplify susceptibility to amphotericin B-related renal and tubular injury, thereby compounding drug-induced injury (55). Consequently, all these findings recommend vigilant monitoring, structured follow-up, and proactive supportive measures to mitigate ADRs and optimize clinical outcomes during amphotericin B therapy (56). All respondents who reported adversities were advised to visit the hospital for follow-up checks and treatment if needed; however, recording revisits was outside our scope. Respondents appreciated the follow-up calls and considered them psychological support during recovery. This indicates the importance of extended support integrating structured telephone-based follow-up into post-discharge care, particularly in resource-constrained settings where access to regular in-person follow-up is limited.

## 5 Strengths and limitations

To the best of our knowledge, this is the first comprehensive analysis of mucormycosis cases in Central India that addresses critical evidence gaps. The study integrates patients’ records with post-discharge MBPD follow-up, enabling continuity of care and tracking post-hospital-discharge outcomes. It includes a detailed audit of antifungal and antibiotic prescribing patterns to support antimicrobial stewardship and follows WHO recommendations. The study provides insights for region-specific clinical guidelines. The inclusion of MBPD follow-up demonstrates a scalable intervention specifically for resource-limited environments.

We also acknowledge the study's limitations. The retrospective component relied on the accuracy and completeness of medical records, which may have led to missing data or underreporting of comorbidities, ADRs, laboratory parameters, and clinical outcomes. The study was conducted at two hospitals in Central India during a defined pandemic period, which may limit generalisability to other healthcare settings or post-pandemic contexts. Patient-reported outcomes may have been affected by memory lapses, survivor bias, or proxy reporting by family members, particularly given the long gap between hospitalization (2021) and follow-up interviews (2023).

Non-response and loss to MBPD follow-up, including unreachability and unanswered calls, may have underestimated post-discharge morbidity and mortality, but this was beyond our control. Therefore, reported adversities and functional outcomes may be overly optimistic and may not reflect the experience of the entire patient population, but this was beyond our control.

The small sample size restricts the generalizability of the results. The Firth-penalized logistic regression findings should be interpreted with caution because of the relatively small sample size and limited statistical power.

The absence of detailed information on surgical management may have been a confounder. We could not adjust multivariably for potential confounders such as disease severity, timing of antifungal initiation, surgical intervention and management, or glycaemic control. This limits causal inference. MBPD follow-up outcome assessments were recall-based and subject to recall and non-response bias.

Finally, antimicrobial stewardship assessments were based on prescription patterns and AWaRe classification, without microbiological confirmation of bacterial coinfections. Despite these limitations, the study provides meaningful real-world evidence on mucormycosis management and survivorship, highlights key gaps in post-discharge care, and demonstrates the feasibility of MBPD follow-up as a scalable surveillance and support strategy in resource-limited settings. The study also provides comparative insights into follow-up methods in similar settings and offers practical guidance for region-specific clinical guidelines.

## 6 Conclusion

The prescription patterns and high adverse outcomes in our study provide crucial information that can help in developing rehabilitation and post-discharge support strategies for the survivors of severe illnesses such as mucormycosis. Antifungals, liposomal amphotericin B and posaconazole, were administered primarily per standard mucormycosis treatment protocols and were linked to deranged laboratory parameters (adverse effects) when prescribed. However, poor pharmacovigilance practices in ADR reporting hinder the identification of the responsible drug and emphasize the need for more robust pharmacovigilance implementation in these settings.

Our study provides a comprehensive overview of the clinical characteristics and treatment outcomes of a cohort of mucormycosis survivors, highlighting the potential of using mobile calls for comprehensive MBPD follow-up and emphasizes the importance of vigilant post-discharge surveillance of adversities. The survivors and family members appreciated MBPD follow-up calls and emphasized the need for structured follow-up, support, and psychosocial care. Our results contribute to a better understanding of the long-term effects of mucormycosis on patients’ daily lives.

The strong association between antifungal administration and adversity suggests that patients requiring antifungal treatment are often exposed to factors that independently increase the risk of adverse outcomes, such as complex clinical conditions, prolonged hospital stays, and exposure to multiple medications and death.

The critical post-discharge outcomes, including mortality, co-infections, particularly with COVID-19, and drug safety considerations, recommend structured, longer-term survivor-centered follow-up care models, especially for patients with known risk factors (steroid exposure, orbital/cerebral spread, uncontrolled diabetes). This will support post-discharge recovery and tackle the complex challenges.

Our MBPD follow-up conducted two years after the pandemic indicates that monitoring should be continued beyond one year, including additional characteristics such as psychosocial well-being, quality of life, functional outcomes, and reconstructive effects. Identifying predictors of long-term morbidity and mortality will be essential to inform targeted interventions and optimize care pathways for mucormycosis survivors within global public health systems.

## Data Availability

The original contributions presented in the study are included in the article/Supplementary Material, further inquiries can be directed to the corresponding author.
