# Supplementary material for: Pharmacological treatment, adversities, and mobile-based post-discharge follow-up of mucormycosis survivors in Central India: strengthening patient care
Source: Front Health Serv. 2026 Oct 15;6:1911647. doi: 10.3389/frhs.2026.1911647 (PMC13716765; doi:10.3389/frhs.2026.1911647)
Supplement: Supplementary file 1 [file Datasheet1.pdf]

Supplementary File 1

MBPD Follow-up Questionnaire for COVID-19 and Mucormycosis (ROCM) Patients

Participant ID code: ______________________
Date of telephonic Interview: ____ / ____ / ______
Interviewer Name: ______________________
Contact Number: ______________________

INTRODUCTION

Namaste. My name is PA, and I am calling on behalf of Hospital, where (Patient name)_____________ was treated for mucormycosis between May-July 2021. We are conducting a study to understand the health, treatment experiences, and recovery of patients who had COVID-19 and/or mucormycosis (black fungus). Your responses will help improve patient care and treatment services.

Your (patient's) participation is completely voluntary. You (patient) may choose not to answer any question or stop the interview at any time. All information shared by you will remain confidential and will be used only for research purposes. The interview will take approximately 10–15 minutes.

CONSENT

QC1. Do you agree to participate in this interview?

☐ Yes (1) → Continue

☐ No (2) → Thank participant and end interview

QC2. Do you permit me to audio-record this conversation for research and quality assurance purposes?

☐ Yes (1)

☐ No (2)

If No, continue the interview without recording. Record the responses on paper instead.

SECTION A: RESPONDENT INFORMATION

A1. Who is providing the information?

☐ Patient (1)

☐ Family Member (2)

☐ No Response (0)

SECTION B: CURRENT HEALTH STATUS

B1. Is the patient completely healthy now?

☐ Yes (1)

☐ No (2)

☐ Death (5)

SECTION C: COVID-19 HISTORY

C1. Did the patient have a COVID-19 infection?

☐ Yes (1)

☐ No (2)

C2. Was the patient given steroids during COVID-19 treatment?

☐ Yes (1)

☐ No (2)

C3. Was the patient given oxygen therapy during COVID-19 treatment?

☐ Yes (1)

☐ No (2)

☐ Unknown (0)

C4(a). In which hospital did the patient receive COVID-19 treatment?

____

C4(b). Specify the type of treatment facility

☐ Government/Public Hospital (1)

☐ Private Hospital (2)

☐ Home Isolation (3)

☐ No History of COVID-19 (0)

C5. How many days did the patient stay in the hospital for COVID-19 treatment? ______ days

C6. Does the patient have any paper/report related to COVID-19 treatment?

☐ Yes (1)

☐ No (2)

C7(a). Was the patient given Remdesivir injection?

☐ Yes (1)

☐ No (2)

☐ Unknown (0)

C7(b). If yes, how many Remdesivir injections were given? _____ injections

C7(c). Were there any side effects after Remdesivir injection?

☐ Yes (1)

☐ No (2)

☐ Unknown (0)

SECTION D: COVID-19 RECOVERY

D1. When did the patient recover from COVID-19? Date: ____ / ____ / ______

OR ☐ Death (5)

D2. How many days after COVID-19 infection was the patient diagnosed with black fungus (mucormycosis)? _____ days

SECTION E: MUCORMYCOSIS (BLACK FUNGUS) TREATMENT

E1. In which hospital was the patient treated for black fungus?

E2(a). Was the patient given Amphotericin B injection?

☐ Yes (1)

☐ No (2)

☐ Do Not Know (0)

E2(b). If yes, from which source were Amphotericin B injections procured?

E3. How many days did the patient stay in hospital for treatment of black fungus? _____ days

E4. Do you have any paper/report related to black fungus treatment?

☐ Yes (1)

☐ No (2)

☐ Unknown (0)

SECTION F: SIDE EFFECTS/ Adversities

F1. Did the patient experience any side effects during or after Amphotericin B treatment or due to black fungus?

☐ Yes (1)

☐ No (2)

F2. Was the doctor informed about these side effects?

☐ Yes (1)

☐ No (2)

F3.Type of Reactions

Reaction	Yes (1)	No (2)
Nausea	☐	☐
Vomiting	☐	☐
Fever	☐	☐
Nephrotoxicity (Kidney Toxicity)	☐	☐
Hypokalemia (Low Potassium)	☐	☐

F4. Any other side effect?

☐ Yes (1)

☐ No (2)

If yes, specify: ____

SECTION G: RECOVERY FROM MUCORMYCOSIS

G1. How many days did it take for the patient to fully recover from black fungus? _____ days

OR ☐ Death (5)

G2. Was there any discomfort after discharge?

☐ Yes (1)

☐ No (2)

☐ Death (5)

If yes, please describe: ____

SECTION H: MEDICATIONS AFTER DISCHARGE

H1. Which medicines were taken after discharge? (Multiple answers possible)

☐ Posaconazole (PO)

☐ Multivitamin (MV)

☐ Other (O)

☐ Death (5)

If Other, specify:

H2. Duration of treatment after discharge _____ days/months

OR ☐ Death (5)

H3. Did the patient complete the full course of medicines?

☐ Yes (Completed full course) (1)

☐ No (Stopped in between) (2)

☐ Death (5)

SECTION I: LONG-TERM OUTCOMES

I1. Did the patient face any financial problems because of the illness or treatment?

☐ Yes (1)

☐ No (2)

☐ Death (5)

I2. Did the patient revisit the hospital for follow-up?

☐ Yes (1)

☐ No (2)

☐ Death (5)

I3. Is the patient still taking any medicine?

☐ Yes (1)

☐ No (2)

☐ Unknown (0)

☐ Death (5)

I4. Has the patient started getting sick repeatedly after recovery?

☐ Yes (1)

☐ No (2)

☐ Death (5)

SECTION J: ADDITIONAL COMMENTS

J1. Is there anything else you would like to tell us regarding the patient's illness, treatment, recovery, or current health condition?

☐ Yes (1)

☐ No (2)

☐ Death (5)

If yes, please specify: ____

Any other comment? ____

____

INTERVIEW COMPLETION

Interview Status

☐ Completed

☐ Partially Completed

☐ Participant Refused

☐ Patient Deceased

☐ Unable to Contact

Thank you very much for taking the time to speak with us today. We sincerely appreciate your participation and the valuable information you have shared. Your responses will help us better understand COVID-19 and mucormycosis and improve care and support for future patients.

Do you have any questions for us before we end the call?

☐ Yes

☐ No

End of Interview.
